# A new approach of microbiome monitoring in the built environment: feasibility analysis of condensation capture

**DOI:** 10.1186/s40168-023-01555-5

**Published:** 2023-06-08

**Authors:** Jarrad T. Hampton-Marcell, Aritra Ghosh, Mohamad Jafari Gukeh, Constantine M. Megaridis

**Affiliations:** 1 Biosciences Division, Argonne National Laboratory, Lemont, IL USA; 2 Department of Biological Sciences, University of Illinois at Chicago, 845 W. Taylor St, Chicago, IL 60607 USA; 3 Department of Mechanical and Industrial Engineering, University of Illinois at Chicago, Chicago, IL USA

## Abstract

**Background:**

Humans emit approximately 30 million microbial cells per hour into their immediate vicinity. However, sampling of aerosolized microbial taxa (aerobiome) remains largely uncharacterized due to the complexity and limitations of sampling techniques, which are highly susceptible to low biomass and rapid sample degradation. Recently, there has been an interest in developing technology that collects naturally occurring water from the atmosphere, even within the built environment. Here, we analyze the feasibility of indoor aerosol condensation collection as a method to capture and analyze the aerobiome.

**Methods:**

Aerosols were collected via condensation or active impingement in a laboratory setting over the course of 8 h. Microbial DNA was extracted from collected samples and sequenced (16S rRNA) to analyze microbial diversity and community composition. Dimensional reduction and multivariate statistics were employed to identify significant (*p* < 0.05) differences in relative abundances of specific microbial taxa observed between the two sampling platforms.

**Results:**

Aerosol condensation capture is highly efficient with a yield greater than 95% when compared to expected values. Compared to air impingement, aerosol condensation showed no significant difference (ANOVA, *p* > 0.05) in microbial diversity. Among identified taxa, Streptophyta and Pseudomonadales comprised approximately 70% of the microbial community composition.

**Conclusion:**

The results suggest that condensation of atmospheric humidity is a suitable method for the capture of airborne microbial taxa reflected by microbial community similarity between devices. Future investigation of aerosol condensation may provide insight into the efficacy and viability of this new tool to investigate airborne microorganisms.

**Importance:**

On average, humans shed approximately 30 million microbial cells each hour into their immediate environment making humans the primary contributor to shaping the microbiome found within the built environment. In addition, recent events have highlighted the importance of understanding how microorganisms within the built environment are aerosolized and dispersed, but more importantly, the lack in development of technology that is capable of actively sampling the ever-changing aerosolized microbiome, i.e., aerobiome. This research highlights the capability of sampling the aerobiome by taking advantage of naturally occurring atmospheric humidity. Our novel approach reproduces the biological content in the atmosphere and can provide insight into the environmental microbiology of indoor spaces.

Video Abstract

**Supplementary Information:**

The online version contains supplementary material available at 10.1186/s40168-023-01555-5.

## Introduction

On average, humans spend approximately 69% of their time inside their residence, 5% at their workplace, and nearly 13% in various built environments; more importantly, humans serve as a main source of microbial matter found within these built environments [1]. Recent studies have demonstrated that humans harbor as many microbial cells as their own and that they emit 30 million microbial cells per hour into their immediate environment [2]. For built environments, the air is one of the primary reservoirs for these aerosolized microbes. But analyzing samples from surfaces entirely misses the time period between the microbiome emission and their “landing.” On the other hand, analyzing samples drawn from indoor air, allows to probe earlier into the released microbiome’s lifetime, having ramifications on our fundamental understanding of the related transport processes. As technology continues to advance and more accurate sampling means are explored, our understanding of the human-built environment interface will continue to expand, along with our ability to track the microbial taxa we emit.

**Fig. 1:**
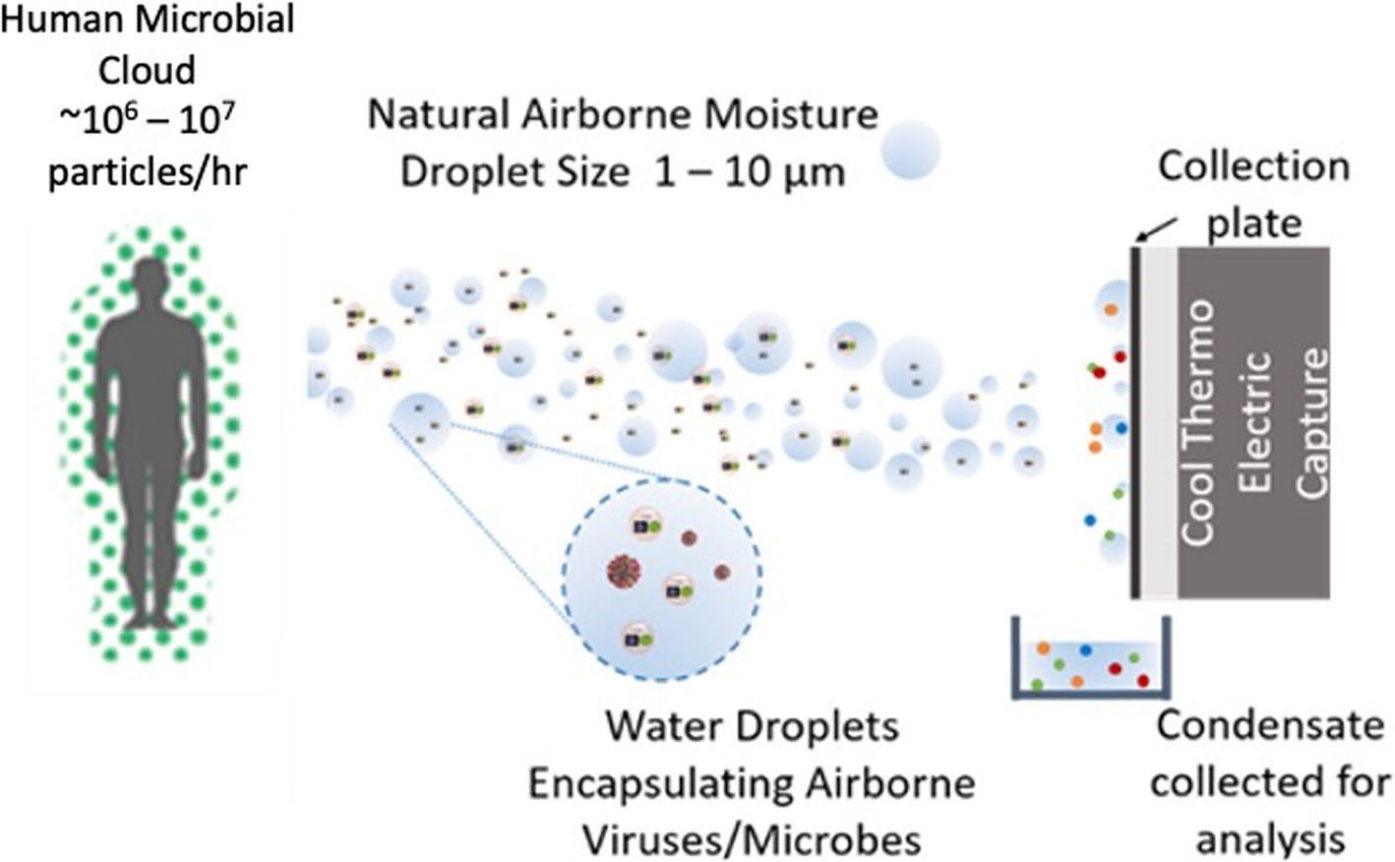
Humans naturally emit 30 million microbial cells per hour, which could combine and get stabilized with water particles naturally found in the humid air surrounding us. These microbial emissions can spread through the air in the built environment. By using a small surface cooled below the dew point, water-dispersed samples can be collected (right), which, in principle, would also contain DNA emitted by the human source. Thus, analysis of the collected condensate can provide a measure of the risk posed by the airborne presence of such particles

Some researchers have characterized the microbiome in the built environment as a microbial wasteland demonstrating that modern sequencing technology is susceptible to low biomass, which is a common dilemma [3]. A major issue in microbiome sampling is the reliability and accuracy of the sample collection approach. Current methods of capturing airborne microbes utilize either passive (settling plates) or active filtration (electrostatic, air impingers), which may damage organisms during collection, which is done in a non-wet state [4]. One approach to potentially improve the collection of microorganisms invokes naturally present atmospheric water, which is known to sustain microbial life and promote reproduction and growth [5]. While aqueous particles naturally exist in the air, few studies have explored the collection of water from the atmosphere in order to analyze the aerosolized microbes that accompany it [6–9]. Previous research has  provided conceptual evidence using aerosol condensation capture to detect targeted microbial identification [10]. However, aerosol condensation capture has yet to be applied to complex communities such as the human and built environment microbiomes. The large numbers of microbes emitted by humans (Fig. 1) can serve not only as a forensic marker of human-built environment interactions but may also provide insight into how airborne microbes (a.k.a. aerobiomes) influence the transmission and microbial community structures found within built environments and their associated human health implications as well.

This study presents the feasibility of a condensation collection device that could be used in series with microfluidic sensors in the future for real-time monitoring of health in the built environment. As sequencing technology continues to improve [11], databases expand [12], and machine learning becomes more refined [13], the dynamic state of the microbial world is destined to become a tool that we can monitor in real time and manipulate to promote human health and hinder disease spread. Our approach uses a cooled metal plate to collect airborne water droplets that also contain aerosolized microbiomes; the cool plate also condenses water vapor naturally present in the atmosphere. The study demonstrates the forensic capability of sourcing microbiomes using this technique, demonstrating it as an emergent tool to elicit host-environment interactions via aerosols.

## Methods

### Study design

For this study, two independent experiments were conducted: (1) theoretical testing of aerosol condensation capture across various sampling times and (2) comparison of aerosol condensation capture to traditional air impingement and the resulting microbial community composition. *Theoretical testing*: Using nuclease-free water, samples were aerosolized for 2–3 h at volumes of 25 ml and 8 h at volumes of 50 ml comparing predicted to expected yields of condensate using Stokes number, which accounts for the behavior of particles suspended in fluid flow. *Microbial characterization*: Air impingers (SKC Leland Legacy pump) were placed at tabletop height in a research laboratory (7200 cubic feet) and an academic building office (1200 cubic feet) and allowed to collect aerosols onto a PTFE filter for up to 24 h. Additionally, a custom-made aerosol condensation capture was placed within 5 ft. of the air impingers to ensure the consistency of collected aerosols. All samples (*n* = 38) were stored at the University of Illinois at Chicago in a − 80 °C until downstream processing at Argonne National Laboratory.

### Theory and validation of condensation capture

Theoretical yields of captured condensate were compared against actual yields using a nebulizer in a closed chamber to validate the accuracy of the method. Under closed-chamber conditions, samples were nebulized and collected on a cooled aluminum plate, thus producing water samples for downstream analysis.

### Aerosol generation and sample capture

**Fig. 2:**
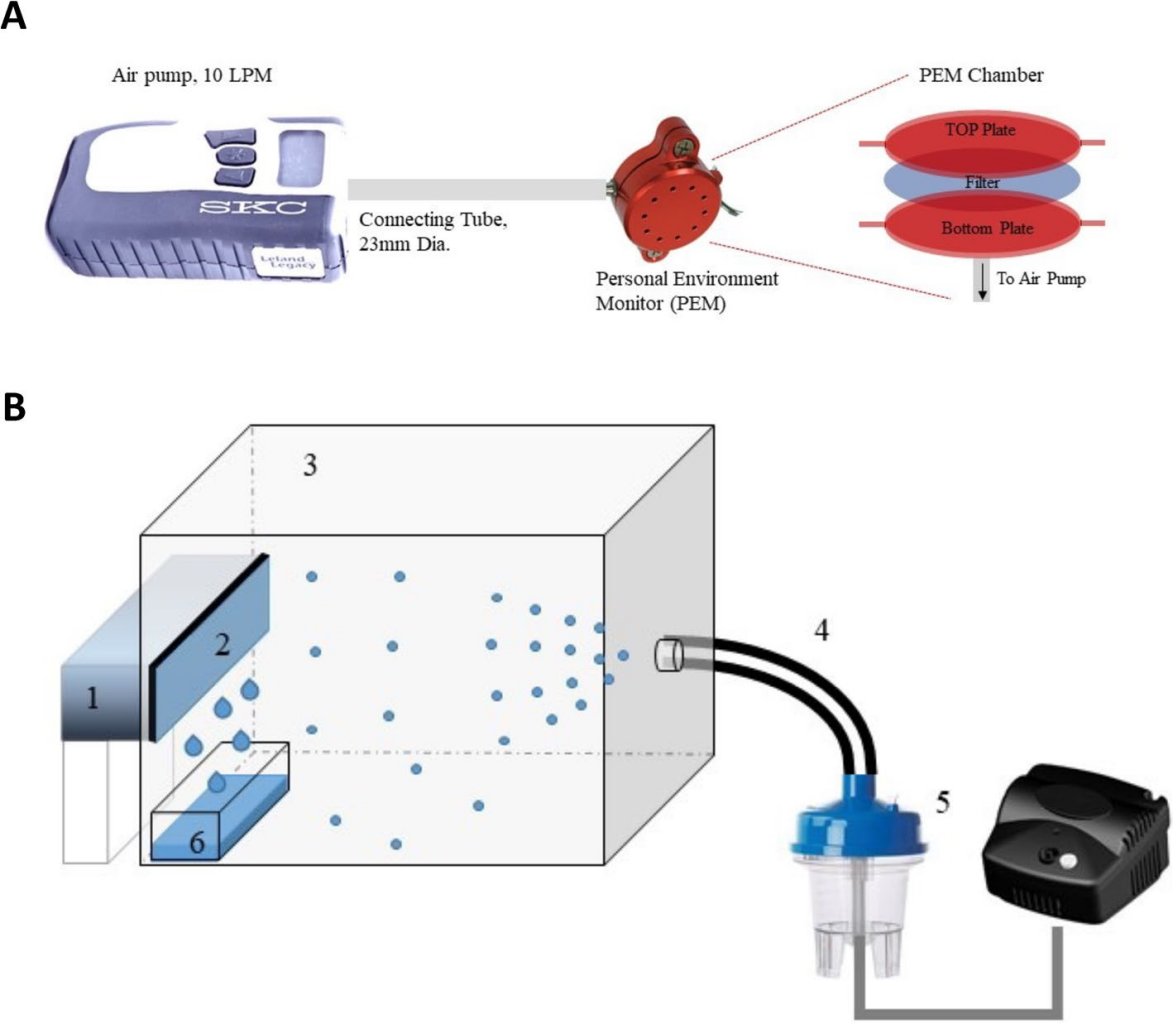
**A** Standard air impinger used to collect dry samples on a filter. The ambient air conditions during the sampling tests were 22 °C and 50% RH. **B** Schematic of aerosolized microbiome collection setup. (1) Peltier cooler, (2) Mirror-finish aluminum plate acting as collection surface, (3) sterilized acrylic box, (4) PVC tubing, (5) nebulizer, and (6) collected condensate. The conditions in the chamber were maintained at 22 °C and 93% RH

Airborne microbes were collected using 2 different setups, both shown in Fig. 2. The first was a traditional air impinger (Fig. 2A), while the second was a custom-made aerosol condensation capture (Fig. 2B), which allowed the introduction of well-defined aerosolized microbiomes into a sterile acrylic chamber [30 cm × 30 cm × 40 cm] and subsequent collection on a cooled sterile metal plate. The air impinger (SKC Leland Legacy pump) drew atmospheric air at 10 ls per minute (LPM), which passed through a PTFE filter (10 µm) contained in a SKC Personal Environmental Monitor (PEM). The system was designed to capture any airborne particles including microbes greater than 10 µm. The filters were weatherized at 35% RH and 20℃ for 24 h on a sterilized petri-dish. After 24 h of airborne sample collection, the used PTFE filters were frozen at − 20℃ to prevent unwanted evaporation from the sample. The condensation setup consisted of a Peltier cooler (condensation platform), the sterilized acrylic chamber, a nebulizer (IV Heart continuous Nebulizer, Westmed) and a pump (Pulmuneb, Devilbiss Healthcare). Before each test, the inner walls of the chamber were covered with food-grade plastic wrap and cleaned by a corona plasma treater (Corona SB) to eliminate organic contaminants. A mirror-finish aluminum plate (McMaster Carr) was used as the collection surface (12.7 cm × 8 cm × 0.3 cm). The 2-cm inner diameter plastic tube connecting the nebulizer to the chamber was rinsed in sterile water and then UV-sterilized for 10 min between uses (unpublished data). The temperature of the cooled plate was maintained at 0 °C. The swabbed microbiome samples collected from the four human subjects were transferred to vials containing sterile water, aerosolized from the vials, and introduced into the chamber from the opposite side of the cooled plate (Fig. 2b). Some droplets were collected on the cooled aluminum plate; the low plate temperature also caused some water vapor (containing no microbiome) to condense on the metal surface. Due to gravity, the collected water dripped down into a small acrylic container placed underneath the vertical plate. After 2–6 h of condensate collection (Table 1), the fluid samples were weighed, poured into sterile vials, sealed, and stored for subsequent microbiome analysis. All portions of the chamber that came into contact with the aerosol were replaced with sterile parts after each experiment to minimize cross-contamination. This process was duplicated for each sample to improve consistency and resolution for downstream analysis.

**Table 1:**
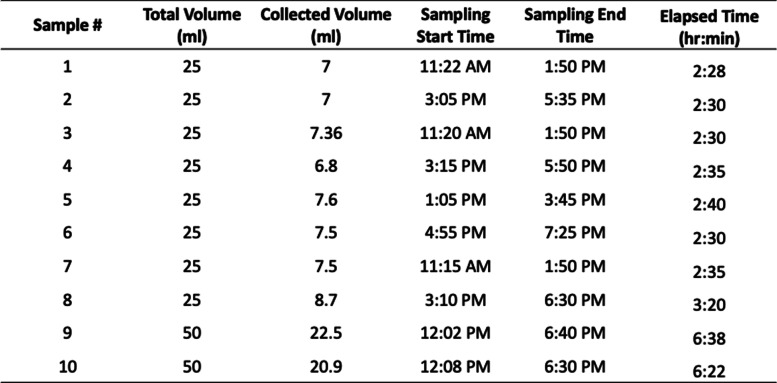
Condensate sample collection details for four healthy human subjects who provided microbiome matter for this work

These tests were conducted in a closed chamber

### Microbiome sample processing and data analysis

Microbial DNA was extracted from the total aerosol volume collected using the air impinger and the condensation setup using the PowerWater DNA Isolation Kit (Qiagen) using the manufactured suggested protocol. DNA was then amplified and sequenced using the Earth Microbiome Project (EMP) suggested protocol [14]. Adapted barcodes for Illumina’s next-generation sequencing platform targeted the V4 region of the 16S rRNA gene (515F-806R). Following PCR, amplicons were quantified using PicoGreen (Invitrogen) and a 96-well fluorescent plate reader. Normalized and cleaned PCR products were pooled and placed onto Illumina’s MiSeq sequencer at Argonne National Laboratory’s Environmental Sample Preparation and Sequencing Facility. Amplicon sequence variants (ASVs) were generated using DeBlur to identify microbial subOTUs, and ASVs were analyzed using Quantitative Insight into Microbial Ecology 2 (QIIME 2) and R Statistical Software [15, 16]. The number of ASVs observed, microbial richness (Inverse Simpson), and evenness (Shannon) were used to measure microbial diversity using the R package “phyloseq.” [17] An analysis of variance (ANOVA) was generated to test whether there were any significant (*p* < 0.05) differences in microbial diversity between sampling platforms. To assess core microbiota, the number of ASVs was measured within each platform, as well as the number of ASVs shared that were present in at least 50% of the samples and had a relative abundance of at least 1% using the R package “ampvis2.” Bray–Curtis dissimilarities were generated to observe differences in microbial community structure, and a permutational analysis of variance (PERMANOVA) was generated to test significance in variation in microbial variation. Random forest models (a machine learning classifier) were generated to assess ASVs that differentiated between normal versus HTN using hierarchical decision trees to identify an ASV’s importance to the model’s accuracy using the R package “randomForest”. All figures were visualized using “ggplot2” [18].

## Results

### Theoretical considerations on aerosol-particle transport

The droplet distribution of the mist generated by the nebulizer has a median value in the range of 2–3 µm (manufacturer-provided values). Assuming that the droplets (diameter *d*_*p*_, density *ρ*_*p*_) are uniformly dispersed in the fog stream entering the chamber with velocity *U* = 3.5 mm/s, the corresponding Stokes number is

$$St=\frac{{\rho }_{p}{d}_{p}^{2}U}{18{\mu }_{g}L}$$

where *µ*_*g*_ denotes the viscosity of the gas and *L* the characteristic length of the collection plate. For the applicable values here, we obtain *St* = O(10^–6^), which suggests that the fog droplets faithfully follow the carrying gas stream. Even if the droplets grow to be much larger due to coagulation in flight, the corresponding *St* would remain much lower than unity, thus confirming that the droplets introduced in the chamber follow the gas flow patterns. After entering the chamber, the particles can settle under the effect of gravity. The characteristic velocity of settling under gravity is given by

$${v}_{s}=\frac{{\rho }_{p}{d}_{p}^{2}g}{18{\mu }_{g}}$$

For the present chamber and according to the above settling velocity estimate, the time required for particles in the size range (2–3 µm) to settle is about 1/3 h; particles close to 10 µm (generated by aerosol coagulation) would settle in a few minutes. Since each sampling experiment lasts for 2–3 h for 25 ml samples and 6 h for 50 ml samples, one would expect a bias in the collection rate if the droplets were not continuously introduced in the chamber, which is not the case here (the chamber is continuously resupplied by the nebulizer). Nonetheless, some collection bias (in terms of droplet numbers) is expected towards the smaller uncoagulated droplets, which stay suspended longer than their larger counterparts, and thus are more likely to reach the plate. However, this bias is countered by the mass dominance of the larger (formed by mid-air collisions) droplets collected on the plate: this translates to the majority of the collected mass sample originating from the larger aerosol drops.

**Fig. 3:**
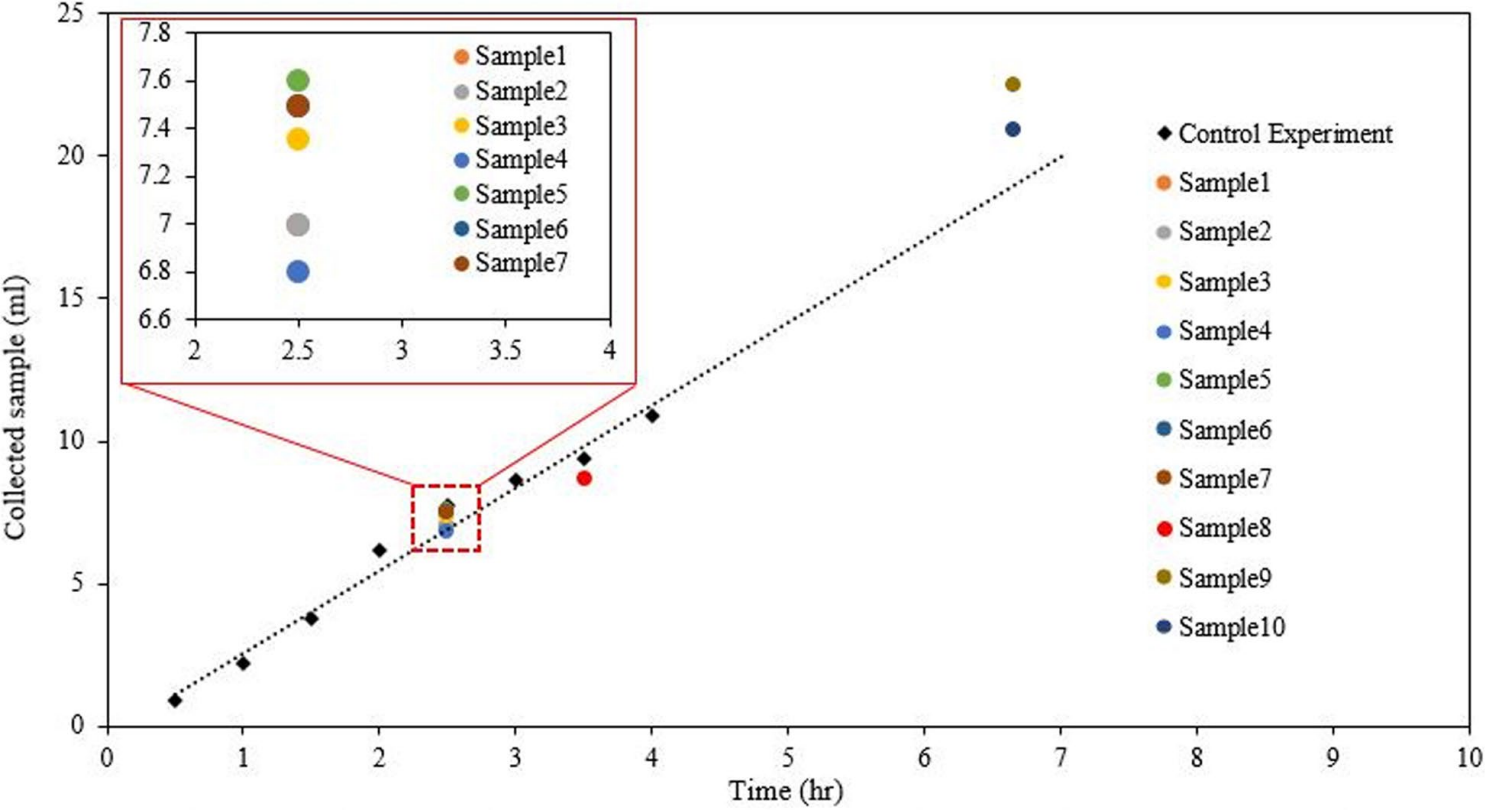
Diamond symbols mark the volume of condensed DI water at time instances ranging from ½ to 4 h from the onset of the control experiment (no microbiome content). The figure also shows the corresponding data for the 10 microbiome samples listed in Table 1 (which also included microbiome content). These samples were collected between 2.5 and 6.5 h after the onset of each experiment. The dash line is a guide for the eye

Using 25 ml of purified water, control samples were aerosolized to test the reliability of condensation capture (Fig. 3). The collection was done over distinct time periods that varied from 0.5 to 4 h after the onset of each experiment. The sample mass collected on the condensation plate from these control experiments showed a linear behavior between collected mass and time passed (Fig. 3). Furthermore, the results for the 10 control samples (Table 1) exhibited consistency between the collected mass in the aerosolized microbiomes and the value expected from the control experiment curve. As seen in Table 1, 27 to 45% of the aerosolized mass sample was collected on the cooled plate during each experiment. Due to the design (fog entry opposite to the collection plate), most of the collected sample came directly from the newly introduced fog which was directed at the plate. Some of the condensates originated from water vapor present in the chamber, which would not influence the microbiome content (the microbiome was present only in the aerosol droplets).

### Microbial condensation sampling compares favorably to air impingement

**Fig. 4:**
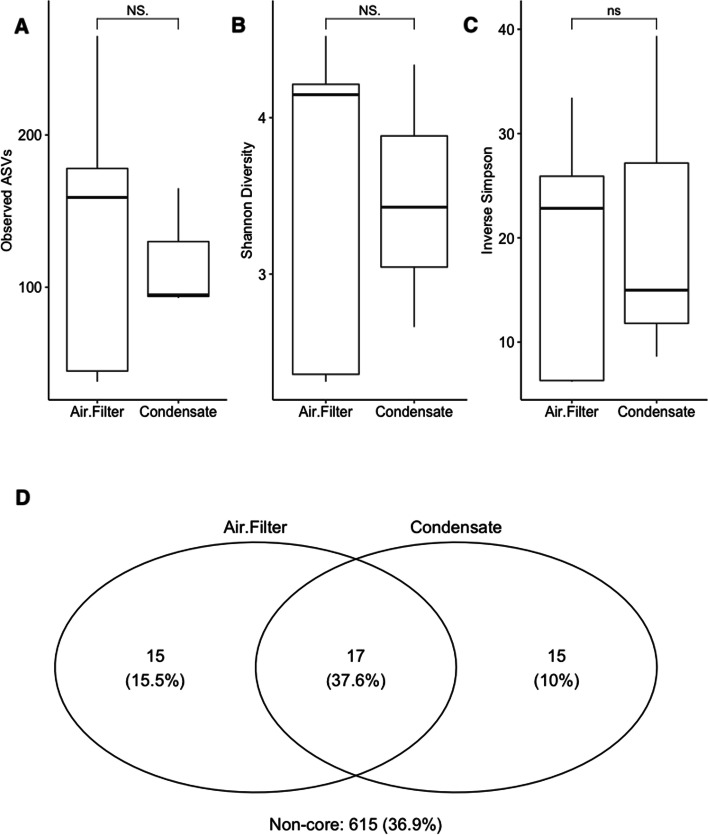
Microbial diversity was measured for samples collected via aerosol impingement and condensation. The number of ASVs (**A**), evenly distributed taxa (**B**), and dominant taxa (**C**) are depicted. The number of shared ASVs, as well as ASVs unique to each air collection platform, are represented as a Venn diagram (**D**)

Following filtering and normalization for this study (*n* = 38), a total of 190,000 sequences encompassing 2561 actual sequence variants (ASVs) were analyzed. To analyze the similarity between aerosol condensation and impingement, microbial diversity, community structure, and composition were analyzed. For alpha diversity measures, condensation capture showed no significant (ANOVA, *p* > 0.05) changes in the number of observed microbial ASVs (Fig. 4A), the number of evenly distributed taxa (Shannon index; Fig. 4B), or the number of dominant taxa (Inverse Simpson index; Fig. 4C) suggesting no differences between the two sampling platforms. Furthermore, Bray–Curtis distances were generated to determine similarity in microbial community structure between platforms, which showed no significant (PERMANOVA, *p* > 0.05) difference either. These findings suggest neither the captured microbial diversity nor microbial community structure are distinctly different between air impingement or aerosol condensation capture. In addition, the number of shared microbial taxa was analyzed, and their microbial relative abundance was at least 10%, found in at least 50% of the samples. 37.6% of the qualifying taxa were shared between platforms, which was greater than the percentage of taxa unique to either aerosol condensation capture (10%) or impingement (15.5%; Fig. 4D).

**Fig. 5:**
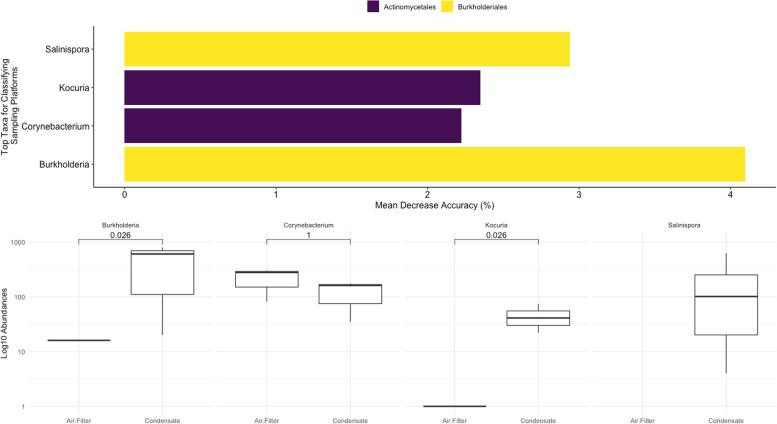
Random forest models were generated to identify microbial taxa differentially abundant between sampling platforms. **A** Feature scores were generated from the random forest model to denote the Top 5 ASVs by plotting the increase in mean error rate following removal from the decision tree. **B** Boxplots were generated to visualize the distribution of ASVs identified by the random forest model and an analysis of variance (ANOVA) was generated to test significance (*p* < 0.05) between sampling platforms

Given sampling platforms were placed in close proximity to one another, it was interesting to observe that approximately one-third of the microbial composition were shared between platform possibly suggesting methods captured differential microbial taxa. When analyzing the microbial composition of aerosol condensation capture, Streptophyta (51.6%) and Pseudomonadales (21.7%) comprised approximately 70% of the microbial sequences (unpublished data). To discern differentially abundant microbial taxa between sampling platforms, random forest models were utilized. Using an out-of-bag model, sampling platforms were moderated and differentiated from one another (AUC = 0.60) with an overall error rate of 37.5% suggesting the two platforms collected microbial taxa that distinguished between the two. To identify microbial taxa important to classifying aerosol condensation capture versus air impingement, feature scores were analyzed which rank microbial importance based on changes in the model’s error rate. Burkholderiales (7.03%) and Actinomycetales (4.57% increased the error rate by 11.61% combined when removed from the training model. Within these two orders, *Burkholderia* (4.09%), *Salinispora* (2.94%), *Kocuria* (2.35%), and *Corynebacterium* (2.22%) were among the most important genera when classifying the two platforms when observing error rates (Fig. 5A). Additionally, distributions of microbial taxa identified by the random forest model were compared between platforms. *Burkholderia*, *Kocuria*, and *Salinispora* abundances were significantly (ANOVA, *p* < 0.05) more abundant via aerosol condensation capture versus air impingement while no significant (ANOVA, *p* > 0.05) differences were observed for *Corynebacterium* suggesting aerosol condensation capture reveals distinct differences in detection of aerosol microbial taxa when compared against air impingement (Fig. 5B).

## Discussion

We have demonstrated that aerosol collection via condensation of natural humidity is a reliable method to investigate the airborne microbiome in the built environment, and under controlled conditions, aerosol condensation is comparable to the common aerosol impingement technique yet captures aerosolized microbial taxa unobserved using air impingement. The latter is the prevailing technique to circulate, filter, and sample the air and its dispersed constituents. However, we must recognize that aerosol impingement is not considered to be the gold standard for analyzing the aerobiome, even though it is commonly employed in practice for studying airborne particulates. Moreover, passive particle settlement on surfaces is also commonly used but not compared in this study. Still, this work highlights the potential to engineer a reusable microbial collection platform that mirrors its predecessors and is efficient and reproducible. These are two aspects that have largely eluded existing platforms. Additionally, collected microbial communities via condensation are likely homogenous, as the sample is collected in an aqueous medium, unlike the dry filters (and thus more inhospitable media) used for aerosol impingement. Thereby, aerosol condensation is believed to generate technical and sampling replicates that increase reproducibility and the ability to perform additional tasks, such as metabolic and proteomic analyses. Through the combination of additional experimental research and refinement in aerosol condensation, it is highly plausible to utilize the present approach to sample open and built environments alike. As our aerosol condensation capture uses equipment readily available and relies on naturally occurring humidity, it serves as a cost-effective and scalable approach to capture aerosolized microbial taxa for both indoor and outdoor environments. Indirectly, it also provides the opportunity to assess microbial dispersion from the outdoor to the indoor environment.

The experimental system used in this work allowed sampling under well-controlled environmental conditions that feature a high level of water content in the air. Naturally, less humid environments (lower RH) would require longer condensate collection times for similar samples. The relative amount of water present in the atmosphere in the form of droplets vs. in vapor form also affects the sampling tests. For example, under the same aerosol feed rate in the chamber, higher RH values would result in larger condensate collection rates, which translates into a higher degree of dilution of the aerobiome in the final sample. Consequently, the sensitivity of the present approach is expected to vary with ambient conditions. This factor is out of the scope of the present study and is left for future works, as are other experimental factors, e.g., chamber size, the relative position of aerosol injection tube and cool plate, air circulation, etc.

Both Psuedomonadales and Streptophyta are commonly associated with outdoor environments, including urban settings, and are typically sourced from plants [19]. Also, it has been suggested that their presence indicates microbial communities that are likely associated with air filtration and ventilation within the built environment, suggesting that the microbial communities captured using condensation are in part a result of HVAC systems filtering and mixing outdoor air into the built environment. Streptophyta usually comprise anywhere between 19 and 45% of the microbial community found in HVAC filters and on surfaces [20, 21]. Additionally, Pseudomonadales is commonly associated with human occupancy and generally deposited on surfaces within the built environment, which is interesting in that we detected the microbial taxa via water droplets collected from indoor air [22]. If aerosol condensation capture is collecting microbial taxa airborne or aerosolized prior to settling, it opens the potential to investigate the spatiotemporal dispersal or targeted and untargeted taxa in the built environment. Interestingly, both Actinomycetales and Burkholderiales are both commonly found in the open and built environment and are commonly associated with aerosolized infections. Multiple species of *Burkholderia* have been associated with the disease making it a public health concern and a target in aerosol detection of potential microbial pathogens. *Burkholderia cenocepacia* infections are often associated with cystic fibrosis [23]. Additionally, *Burkholderia pseudomallei* is commonly found in soil and commonly associated with aerosol infections making it a public health concern. Previous studies have shown that *B.* *pseudomallei* aerosol detection is likely dependent on the sampler’s capacity to protect the sample from desiccation [24]. This would likely favor aerosol condensation capture over air impingement as *Burkholderia* is captured in its existing aqueous environment likely making it a less disruptive method consequently reducing DNA degradation. More importantly, aerosol condensation capture may provide a unique advantage to elicit the spread of potentially aerosolized pathogens, which would be beneficial in cases of hospital settings, nursing homes, and other environmental settings where an individual’s health may be compromised. As active detection and mitigation of potentially infectious organisms are of the utmost importance in these situations, future studies could be employed to test efficacy of real time detection using condensation capture, as well as the viability of microorganisms captured.

While further investigations could provide added insight, this study analyzed neither the sensitivity/specificity of aerosol condensation capture nor the viability of microorganisms collected. As it currently stands, we are unsure how our approach compares to similar aerosol condensation capture platforms or passive/active collection of “wet” samples [6, 10]. Also, it is not clear how effective aerosol condensation is at capturing aerosolized or airborne content at very low concentrations. Microbial loads (colony form units per unit volume) have been observed to be anywhere between 400 CFU/m^3^ and 8000 CFU/m^3^ in built environments [25, 26]. Given that condensation capture collects microbes that naturally coexist in water droplets, it is plausible that the technique might be suitable for studying the viable aerobiome. Future studies should explore these aspects of aerosol condensation and its potential to analyze the aerobiome and its dynamics. Specifically, it would be beneficial to elicit whether aerosol condensation consistently captures microbial communities at low microbial loads, especially at ranges where biomass nears the minimum threshold for detection using traditional passive and active techniques. Often, lengthy collection times are required to resolve these conditions, which may mask microbes of interest. Additionally, future studies should elicit whether aerosol condensation increases the chances of analyzing the number of viable microbial taxa collected, which would provide much-needed insight into the bioactivity of the microbiome in the built environment even though it is considered a dessert [3].

## Conclusion

This study highlights the potential to use aerosol condensation capture as a suitable method to measure microbial community composition and structure in indoor air. Because the aforementioned technique collects and condenses naturally occurring water droplets in the air, it makes for an interesting approach to study the dispersion and distribution of the aerobiome. In addition, when one envisions the in-series incorporation of the present technique with microfluidic sensing devices, the potential for a new method that can follow the microbiome population dynamics and its advantages in terms of monitoring sensitive indoor settings becomes apparent. Nevertheless, it remains unclear whether aerosol condensation is suited for all environments where microbial load and humidity vary dynamically, and future studies should look to elicit these underlying associations. In a larger context, aerosol condensation capture may provide further insight about the shortcomings that have continued to elude researchers regarding the spatiotemporal relationship between the microbiome and the built environment.

## Data Availability

All sequence data and associated metadata have been made available through Figshare and are available upon request.

## Abbreviations

ASV: Actual sequence variant
ANOVA: Analysis of variance
PERMANOVA: Permutational analysis of variance
